# Supplementary material for: Are carbon emissions trading and green financial instruments synergistic? -Comprehensive quantitative research based on content analysis
Source: PLoS One. 2024 Mar 7;19(3):e0298601. doi: 10.1371/journal.pone.0298601 (PMC10919601; doi:10.1371/journal.pone.0298601)
Supplement: S1 Data — (ZIP) [file pone.0298601.s001.zip › Data/地级市Z_F工作报告.zip!/╜¡╦╒/╨∞╓▌╩╨/└┤╫╘╣1⁄2╓┌║┼í╛┬φ┐╦╩2╛▌═°í┐_2010.docx]

各位代表：

　　现在，我代表市人民政府向大会作工作报告，请予审议，并请市政协各位委员和其他列席人员提出意见。

　　一、2009年工作回顾

　　2009年，受国际金融危机影响，我市经济社会发展经受了新世纪以来最为严峻的挑战和考验。在省委、省政府和市委的正确领导下，全市广大干部群众深入学习实践科学发展观，以振兴徐州老工业基地为主线，认真贯彻落实中央扩大内需的决策部署，全力以赴保增长、保民生、保稳定，较好地完成了十四届人大二次会议确定的各项任务。

　　（一）综合经济实力不断增强

　　经济实现平稳较快增长。全年完成地区生产总值2220亿元（预计，下同）、增长13.5%以上；实现财政总收入（含基金）467.6亿元、增长30%，一般预算收入164.3亿元、增长30.6%；金融机构各项存款余额2172.6亿元、比年初增加453.5亿元，各项贷款余额1132.6亿元、比年初增加336亿元；城市居民人均可支配收入和农民人均纯收入分别达18800元、6900元，均增长11%。

　　投资和消费需求持续扩大。“三重一大”项目进展顺利，全社会固定资产投资完成1600亿元、增长28%；其中工业投资820亿元，超过全社会固定资产投资的一半；“四大产业”完成投资860亿元、增长32%。消费拉动作用明显增强，全社会消费品零售总额达800亿元、增长18%，消费对gdp增长贡献率达44%。

　　产业升级步伐不断加快。四大主导产业培育成效显著，装备制造、能源、食品及农副产品加工三大产业实现产值2390亿元、增长22.8%，商贸物流旅游业实现营业收入1288亿元、增长17.8%。徐工集团营业收入突破500亿元，“中国工程机械之都”通过国家认定。中能多晶硅产量达7500吨、跃居亚洲第一。高新技术产业完成产值519亿元、增长51.6%；全市开发市级以上新产品1500项，专利申请量达6900件、授权量达2400件。服务业实现增加值840亿元，增长15.5%；浦发、莱商等商业银行来徐设立分支机构,徐州经济开发区获批省级国际服务外包示范区。

　　（二）改革开放扎实推进

　　重点领域改革稳步实施。市县政府机构改革和乡镇综合改革启动，362家国企改革全面完成，市直经营性文化事业单位和文艺院团转企改制有序实施，铜山县、云龙区基本药物零差率销售试点稳步推进，市级投融资平台建设进一步强化，农村新型金融组织建设加快，新沂市被评为“江苏省金融生态示范县（市）”。民营经济快速发展，民营工业产值增速高于全市工业11个百分点。

　　开放型经济加快发展。全市实际到帐注册外资6.9亿美元、增长19.8%，增幅列全省第三；全年共引进118名高层次创新创业人才。自营出口完成15.2亿美元，下滑趋势得到基本遏制；新签外经合同额、完成营业额分别增长39%和35%。大力加强开发区建设，徐州经济开发区和其他7个省级开发区实现业务总收入2368亿元、增长50%。南北共建园区建设扎实推进，东方鲁尔工业园列入国家商务部指导项目。

　　（三）统筹发展进一步加快

　　现代农业发展和新农村建设扎实推进。粮食总产实现六年连续增产，高效农业占耕地总面积比例、设施农业总面积、高效农业产值继续保持全省第一。市级以上农业产业化龙头企业实现销售收入241亿元、增长15.5%；农民专业合作社发展到2271个，入社农户达68.1万户。重点规划建设40个中心镇，新建成50个市级新农村示范村和200个环境综合整治示范村，解决了100万农村人口的饮水安全问题。

　　县域经济发展速度明显提高。铜山工程机械和钢铁冶金、沛县煤化工和铝加工、丰县盐化工和电动车、邳州板材和家具、新沂农用和精细化工、睢宁白色家电和纺织等特色产业规模层次稳步提升。六县（市）工业增加值增长22.3%，高于全市4.5个百分点，铜山、邳州跨入全国百强县行列。

　　节能减排和生态建设成效显著。全市万元gdp能耗下降5.8%，化学需氧量、二氧化硫排放量完成年度省定任务。全部关闭水泥立窑生产线，建成城镇污水处理厂17座，南水北调截污导流工程投资量完成过半。实施了9257亩市区山地绿化和15项绿化景观重点工程，全市森林覆盖率达27.6%，市区建成区绿化覆盖率达41.6%。

　　各项社会事业协调发展。全市80%的小学和初中建成市标准化学校，投入2.67亿元实施“中小学校舍安全”等工程建设，义务教育学校绩效工资全面落实，职业教育和高等教育加快发展。中国矿大科技园二期工程如期竣工，动漫博物馆建成开放，镇文化站和行政村“农家书屋”投入使用，徐州剪纸被联合国教科文组织列入“人类非物质文化遗产代表作名录”。城乡公共卫生服务体系日趋完善，9大类22项基本公共卫生项目实现全覆盖。人口和计划生育工作进一步加强，人口自然增长率控制在6‰以下。全民健身活动深入开展，14人次获世界冠军和全运会冠军，成功申办省第18届运动会。全力支持四川灾后重建，援建工作全省领先。人防、民兵、民族宗教、双拥、档案、外事、侨务、台湾事务、统计、物价、广播电视、新闻出版、住房公积金管理、无线电管理、淮塔管理、气象、妇女儿童、青少年、关心下一代、老龄、扶贫等各项事业都取得了新的进展。

　　（四）中心城市建设力度显著加大

　　综合交通枢纽地位进一步巩固。京沪高铁徐州段线下工程顺利完成，徐连铁路电气化改造和火车站风雨棚改造基本结束，丰沛铁路全面开工，徐济高速江苏段建设加快推进，观音机场国家一类对外开放口岸建设、大郭庄机场搬迁和徐兰客运专线项目有力推进，徐州港总体规划获部、省联合批复，徐淮铁路和徐连城际铁路纳入省政府长三角铁路网规划。

　　功能性项目建设加快推进。市区12条道路改扩建及7个路口渠化改造工程竣工，珠江路和徐贾快速通道按计划推进，云龙湖“四合一”工程、规划馆、档案馆等项目建成开放。中心商圈、新城区商圈和高铁站区商圈建设全面展开，彭城壹号和南湖水街等月光经济区竣工运营。新城区公共服务设施建设进展顺利，工程学院新校区一期工程投入使用，行政办公区基本建成。

　　城市管理水平不断提高。积极构建“大城管”体制，基本形成部门协同、齐抓共管的格局。对12个马路市场和7个城市出入口等进行专项环境整治，对32条道路户外店招实施改造，设置规范便民服务点470余处，主城区保洁市场化管理实现全覆盖，开放型公园和景区管理机制逐步完善，文明城市创建活动深入开展。

　　（五）民生改善取得新成效

　　重大民生问题有序解决。8大类为民办实事项目基本完成。集中力量解决振兴老工业基地三大历史遗留问题，制定并实施棚户区改造计划，出台了拆迁补偿安置、审批手续简化和规费减免等政策措施，完成棚户区拆迁面积175万平方米，建设和购买安置房180万平方米，对41个老居民小区进行了综合整治；采煤塌陷地复垦治理完成3万余亩，置换指标分配使用，中德合作徐州生态示范区建设取得积极进展；采煤塌陷区村庄搬迁工作稳步推进，矿地关系和谐稳定。

　　就业创业工作扎实推进。突出抓好“政策促就业、培训促就业、创业促就业”工程，培训各类技能就业人员26.2万人次，发放创业担保贷款突破1亿元，开辟了大学生创业培训基地，我市荣获首届“中国创业之城”称号。全年城镇新增就业11.8万人，下岗失业人员再就业5.6万人，新增农村劳动力转移就业9.16万人。

　　社会保障体系逐步完善。全市社会保障支出88.78亿元、增长20.4%。企业职工社会保险覆盖面进一步扩大，城镇居民医疗保险参保率达95.5%，新型农村合作医疗参合率达99%。弱势群体基本生活保障水平不断提高，在省内率先建成残疾人基础数据库，为5.7万名就业困难人员发放社保补贴11239.7万元；城乡低保标准分别提高到每人每月283元和130元；发放灾害救助资金2600万元；新建经济适用房9220套，新增廉租住房1280套，发放廉租住房租赁补贴1238万元。

　　和谐稳定的社会局面?包案责任制，较好解决了一批信访突出问题。应急管理体制机制逐步完善，安全生产和食品药品安全工作不断强化，“平安徐州”、“法治徐州”建设深入推进，确保了社会大局稳定。

　　（六）行政效能明显提升

　　行政审批制度改革继续深化。审批事项削减幅度达39%，部门行政审批职能实现相对集中，市行政服务中心许可事项入驻率和窗口现场办结率分别达88%和82%。取消、合并市级行政事业性收费项目29项，降低收费标准27项，减轻企业和社会负担5000万元。

　　“勤廉徐州”创建活动扎实开展。继续开展万人评议机关活动，加大行政问责力度，强化层级监督、行政监察和审计监督，绩效考核体系逐步完善。行政权力网上公开透明运行和电子监察系统开通使用，建立完善了重大决策、重要部署和重点工作的跟踪督查制度及责任追究制。严格落实党风廉政建设责任制和行政执法责任制，自觉接受市人大及其常委会的法律监督和工作监督，主动接受市政协及社会各界的民主监督，以及社会公众和新闻媒体的监督，依法行政、廉洁从政水平进一步提高。

　　在充分肯定成绩的同时，我们也清醒地认识到，当前我市经济社会发展中还存在一些突出矛盾和问题：农业发展基础仍较薄弱，农产品精深加工和流通体系建设相对较慢，农民收入持续增长的难度进一步增加。工业经济增长特别是部分骨干企业的生产经营还面临不少困难，高新技术产业比重不高。实际利用外资总量偏小，出口形势依然严峻。财政收支矛盾比较突出，城乡居民就业、社会保障、看病、上学等问题仍然较多，城市交通拥挤问题日益突出，维护社会稳定的任务较为繁重。一些政府部门效率不高，少数干部服务意识不强，个别干部还存在腐败行为等。对于这些问题，我们将在今后工作中重点加以解决。

　　各位代表，回顾过去一年的工作，在复杂的形势下我市取得的成绩来之不易。这是全市上下认真落实中央和省委、省政府的决策部署及一系列政策措施，坚持迎难而进、全力推动发展的结果，是各位人大代表、政协委员和社会各界人士监督、支持与帮助的结果。在此，我代表市人民政府，向全市各族人民群众、人民解放军驻徐部队、武警官兵和公安民警，向各民主党派、无党派人士、工商联和人民团体，向支持徐州现代化建设的香港特别行政区同胞、澳门特别行政区同胞、台湾同胞、海外侨胞和各界人士，向投身徐州经济社会发展的国内外投资者和企业家，表示衷心的感谢并致以崇高的敬意！

　　二、2010年经济社会发展总体思路及主要目标

　　今年是实施“十一五”规划的最后一年，也是振兴徐州老工业基地的关键一年，做好今年的工作具有特别重要的意义。综合分析今年的宏观经济环境，总体趋势向好，机遇与挑战并存。从国际看，全球经济复苏与诸多制约因素相互交织，主要经济体和新兴市场国家经济运行的积极信号明显增加，但不确定因素依然较多，金融危机影响尚未根本消除，贸易保护主义加剧，低碳经济将带来新的挑战。从国内看，政策机遇延续与结构调整紧密结合，国家今年将继续实施积极的财政政策和适度宽松的货币政策，把调结构、防通胀、促民生作为工作重点，既为推动经济结构转型升级提供了政策支持，也对我们的工作提出了更高要求。从省内看，各市之间的竞争态势日趋明显，在苏南产业结构升级、沿海开发起步、周边城市竞相发展的大背景下，我市面临前有进位机遇、后有赶超压力的竞争环境。对此，我们要牢固树立忧患意识，以更大的力度推动又好又快发展。

　　今年政府工作的总体思路是：以邓小平理论和“三个代表”重要思想为指导，全面贯彻党的十七大和十七届三中、四中全会精神，深入落实科学发展观，按照中央提出的“五个更加注重”的要求，牢牢抓住振兴徐州老工业基地、江苏沿海开发和融入长三角三大机遇，进一步解放思想、扎实工作，着力加快产业结构调整，着力做大做强区域性中心城市，着力强化改革创新，着力改善民生与建设和谐社会，推动经济社会发展再上新台阶。

　　今年国民经济和社会发展的主要预期目标是：（1）地区生产总值增长13.5%。（2）财政一般预算收入增长18%。（3）全社会固定资产投资增长26%。（4）实际到帐注册外资增长15%。（5）自营出口总额增长14%。（6）社会消费品零售总额增长18%。（7）城市居民人均可支配收入增长10%，农民人均纯收入增长10%。（8）城镇登记失业率控制在4%以内。（9）高新技术产业产值占规模以上工业的比重达15%，科技研发投入占gdp比重达1.45%。（10）单位地区生产总值能耗下降5.5%。（11）化学需氧量和二氧化硫排放量完成省下达的减排任务。（12）人口自然增长率控制在6‰以内。

　　围绕实现上述目标，我们将着重处理好五个关系：一是处理好新型工业化与城市现代化良性互动的关系。坚定不移地实施新型工业化第一方略，注重运用工业化理念发展农业和现代服务业，把加快城市化进程作为拉动增长、优化结构、扩大消费的突破口，同步推进产业发展与城市建设。二是处理好传统产业与新兴产业协调发展的关系。把保增长与调结构紧密结合，认真贯彻国家关于产业升级和新兴产业发展的各项规划，加快发展四大主导产业，实施高新技术产业跨越发展、传统产业加速调整、创新型经济培育、农业提档升级“四大行动计划”。三是处理好招商引资与全民创业双轮驱动的关系。牢固树立抓项目就是抓发展的理念，强化专业招商队伍建设，明确招商引资主攻方向，健全招商激励机制，形成招商引资的强大合力；大力营造全民创业的浓厚氛围，深度挖掘全民创业潜力，加快形成内源型和外源型经济齐头并进的发展格局。四是处理好争先进位与和谐建设统筹兼顾的关系。按照发展是第一要务、稳定是第一责任的要求，全面开展对标找差活动，确保主要经济指标增长快于全省、快于上年、快于小康进程，强力提升全市和各县（市）区综合实力在全省的位次；积极稳妥推进各项改革，切实做好民生改善工作，加快解决振兴老工业基地三大历史遗留问题。五是处理好重点带动与整体推进点面结合的关系。把重大产业、重大基础设施、重大城建和实事工程项目以及事关改革发展稳定的大事作为各项工作的重中之重，集中力量抓好130项重大项目建设，确保全社会固定资产投资突破2000亿元、工业投入突破1000亿元，为保增长、调结构、促发展提供强有力的支撑。

　　三、全力推进经济又好又快发展

　　徐州老工业基地振兴最根本的任务是加快产业振兴。我们将加大经济结构调整力度，深入推进改革开放和自主创新，大力发展民营经济和县域经济，不断提升经济增长的质量和效益。

　　（一）扎实做好“三农”工作。认真贯彻中央农村工作会议精神，全面落实强农惠农政策，加快完善现代农业发展体系，进一步强化农业基础、稳定农业生产、促进农民增收。一是切实稳定粮食生产。深入实施农业新品种、新技术、新模式工程，推进粮食高产创建和高效农业规模化，加强高标准农田和水利工程建设，进一步改善农业抗灾防灾条件，巩固和提升粮食生产能力。二是大力发展现代农业。实施“2020”工程，重点建设20个万亩连片的设施蔬菜、瓜果生产基地，20个大中型畜禽和水产养殖场。加快现代农业园区和农产品加工区建设，积极创建国家级农业示范园。着力培育引进一批农副产品深加工企业，发展壮大林果、生态肉鸭、奶牛、大蒜四大产业化体系。积极开展“学寿光、赶寿光”活动，加大农产品品牌创建力度，大力拓展农产品销售渠道。加强农村专业合作组织和农民经纪人队伍建设，力争40%以上的农户加入农村专业合作组织。三是努力增加农民收入。继续采取多种措施促进农民增收，把促进农民就业创业作为最大的农民致富工程来抓，加强技能培训，拓展外出就业空间，促进就地就近就业；鼓励发展多种形式的村级集体经济，帮助农民从农业生产、农产品加工和市场流通中增加收入。四是深入推进新农村建设。加强农村基础设施、生态环境和治安环境建设，完善农村综合服务体系，继续办好“农村六件实事”，加大扶贫开发力度，新建50个市级新农村示范村和200个环境整治示范村，完成20万户农村改厕任务，解决好70万农村人口的饮水安全问题，实现27万人脱贫目标。五是深化农村各项改革。稳定和完善农村土地基本经营制度，加快推进土地承包经营权流转，发展多种形式的农业适度规模经营，拓宽农民增收渠道。加快农村金融改革，积极发展农村小额贷款公司、村镇银行和金融互助组织，建立健全农业担保体系。深化集体林权制度改革，基本完成主体改革任务。

　　（二）加快工业结构调整步伐。坚持“做强主导产业、做新传统产业、做大新兴产业”的结构调整思路，加快提升工业经济发展层次。一是强化主导产业链条延伸。装备制造业重点延伸工程机械主机和关键零部件产业链，壮大矿山、建材、锻压设备和专用车辆等特色装备制造业，推进徐工斗山柴油发动机等项目建设，进一步巩固和提升“中国工程机械之都”的地位。能源产业重点延伸多晶硅－太阳能电池－电池组件－太阳能发电系统等光伏产业链，实施年产9000吨电子级多晶硅、硅棒切片等项目。食品及农副产品加工业重点延伸精深加工产业链，大力发展乳品及饮料、休闲食品及烟草制品业，重点建设维维食品产业园、徐烟技改以及肉鸭、水产和??着力培育形成煤焦化、甲醇深加工、氯碱和精??型材和再生铝为终端产品的铝产品精深加工产业链，推进骨干钢铁企业升级改造；加快建设日产万吨水泥熟料生产线，延伸新型建材、特种板材及高档家具产业链。三是强化新兴产业倍增发展。重点壮大新医药、新材料、电子信息、节能环保和物联网等新兴产业，加快建设恩华药业原料药基地等新医药项目，瑞源科技镭射定转纸等新材料项目，台湾荧茂触摸屏及保护镜片等电子信息项目，高创风电设备等节能环保项目，力争新兴产业规模两年翻番。

　　（三）打造现代服务业高地。坚持生产性服务业和生活性服务业互动并进，促进服务业发展提速、比重提高、结构优化，加快建设一批现代服务业集聚区。一是重点壮大商贸物流旅游业。加快力宝购物中心、苏宁综合商务广场、中央国际商务广场、金鹰国际二期等中心商圈项目建设，有序推进世茂商业步行街、老东门时尚街区、中华老字号精品街区、西部中介商业街等商业街区建设；继续抓好徐州经济开发区生产物流、新城区生活物流、九里综合物流等五大物流基地建设；精心编制全市旅游业发展总体规划，加快推进主题公园、吕梁山风景区、大洞山风景区和窑湾古镇等一批旅游项目建设。二是大力发展总部经济、动漫软件等新兴服务业。发挥城市中心区商务楼宇相对集中和高铁枢纽即将形成的优势，重点发展总部经济、服务外包、金融保险等服务业。大力发展动漫创意、软件开发、科技研发、教育培训等创意产业和技术服务业，加快徐州软件园、徐州经济开发区软件及服务外包基地、创意产业园等项目建设。三是着力增强消费对经济增长的拉动作用。继续拓展城市社区商业服务领域，年内新开40家社区便民店。健全农村消费品流通体系，力争镇村两级农家店覆盖面分别达到100%和92%以上，新增农业生产资料销售网点105个；做好家电、汽车摩托车下乡和家电以旧换新工作，充分释放农村消费市场的潜力。

　　（四）促进民营经济和县域经济加快发展。坚持因地制宜、分类指导，做大民营经济，做强县域经济。一方面，加速民营经济崛起。完善促进民营经济发展的政策措施，加快构建公平竞争平台，进一步降低民营企业准入门槛，鼓励民间资本进入基础设施、公用事业等领域。切实规范涉企检查行为，推行收费公示和企业付费登记卡制度。继续推行中小企业金融顾问制度，着力解决民营企业融资难题。抓好重点骨干企业提档升级和新增长点培育，鼓励支持民营经济产学研联合，帮助有潜力的企业加快上市融资。另一方面，加快县域经济发展。完善县域经济发展综合考核体系，深入开展比学赶超活动，营造争先进位的浓厚氛围。加大县域产业结构调整力度，力争各县（市）每年新上一批亿元以上项目，推动重点产业集群集约发展。积极争取国家和省加强中小城市及小城镇发展的扶持政策，放宽县城镇落户条件，培育壮大一批工业强镇、旅游强镇和商贸强镇，进一步提高各地城镇化水平。加强各县（市）金融生态环境建设，组建市、县、镇三级担保机构及商业性、互助性担保机构。继续实施大学生村官工程，推动高层次经营管理人才和专业技术人才投身县域经济发展。采取有力措施，促进贾汪区经济加快发展。

　　（五）努力提高开放型经济发展水平。大力实施开放带动战略，力争实现开放型经济发展新突破。一是构建招商引资新格局。完善招商引资体制机制，强化县（市）区和开发区的招商主体地位，根据市相关部门职能落实招商责任，提高考核权重，加大奖惩力度。加强专业招商队伍建设，强化招商人员技能培训和绩效挂钩。突出招商重点，围绕四大产业和“三重一大”，加强对台对港对德以及央企和民企的招商，精心办好各类招商活动。高度重视招才引智工作，强化高层次人才引进和载体建设。二是提升开发区的承载力和竞争力。重点强化徐州经济开发区建设，组织开展“产业升级年”活动，推进国家级开发区申报、国家级出口加工区创建工作，积极带动徐州工业园发展，加速迈进全省先进开发区行列。建立完善其他省级开发区和南北共建园区的激励考核机制，优化乡镇招商引资统计考核办法，推动招商引资项目向开发区集中。三是加强出口基地和品牌建设。加大对骨干外贸企业的扶持力度，狠抓工程机械、新能源、优质农产品等重点产品出口，着力引进一批以出口为主的利用外资项目。积极实施“走出去”战略，鼓励冶金、板材等优势企业赴境外投资和组建营销网络，扩大国际市场份额。

　　（六）切实抓好环境保护和土地节约集约利用。不断加大环境和土地的保护力度，强化环境改善和土地永续利用。加强环境治理和生态建设。编制实施流域水环境整治规划，健全突发环境事件应急处置机制。强化节能减排目标责任制，努力发展低碳经济，加大小造纸、小焦化等“五小”企业生产线关闭力度，坚决杜绝新上高耗能高污染项目；扎实推进淮河流域水污染防治项目建设，全面完成省下达的目标任务。逐步推进城区化工企业搬迁，加强主城区北部环境综合整治，市区建成和改造5个污水处理厂。组织开展重点企业和公共机构节能行动。启动实施国家森林城市和生态园林城市创建工作，抓好吕梁山景区山地绿化等生态环境项目建设。强化节约集约用地。坚持最严格的耕地保护制度和最严格的节约用地制度，按照“质量优先、规模优先、急用优先、集约优先”的原则，切实提高用地门槛和投资强度，重点保障符合规划和产业政策、科技含量高、投资密度大、成长性好的项目用地。充分利用增减挂钩、万顷良田建设和振兴老工业基地等政策，加快推进土地整理和采煤塌陷地治理，加大闲置土地清理处置力度，积极拓展用地空间。把节约集约和依法规范用地工作纳入县（市）区综合考核，完善土地违法责任追究和执法奖惩机制，坚决遏制违法违规用地行为。

　　（七）加大改革创新推进力度。围绕增活力、强动力，抓住改革和创新两个关键环节，努力解决制约发展的深层次问题。精心组织实施各项改革。全面完成市县政府机构改革，建立有机统一的大部门管理体制，上半年基本完成乡镇机构改革。加快事业单位改革，重点抓好生产经营类事业单位改企转制，统筹推进行政管理执行类、社会公益类事业单位改革，实施事业单位绩效工资，建立事业单位岗位设置管理制度。继续深化国有企业改革，完善企业内部法人治理结构。健全财政部门预算和国库集中支付制度，建立市风险投资公司，整合市级投融资平台，继续大力引进外地股份制金融机构。优化整合文化产业资源，成立文化产业发展集团和演艺集团。深化医药卫生体制改革，实施基本药物制度，推动公立医院改革试点，建立医保异地就医结算平台。加快培育创新型经济。推进中国矿大科技园、省“333”工程科技成果转化徐州基地、海外科技人才创业基地、国家级留学生创业园等科技创新公共服务平台建设，力争工程机械研究院、工程机械研发试验检测中心、多晶硅工程中心建设取得新突破。大力实施知识产权战略，组织对关键项目和核心技术的联合攻关，争取年内全市专利申请量达7000件、授权专利2500件。鼓励引导企业增加研发投入、创建自主品牌，强化科技成果转化应用，建立“政产学研金”合作联盟。加快实施“徐工产品智能化工程”等一批信息化示范项目，重点建设一批信息化示范企业。

　　四、突出抓好特大型区域性中心城市建设

　　大力实施23项重大基础设施项目和8大类149项城建重点工程，进一步完善综合服务功能，整体提高中心城市的辐射带动能力。

　　（一）强化重大基础设施建设。扎实推进京沪高铁徐州段、丰沛铁路、徐济高速、宿新高速、观音机场改扩建、南水北调等续建项目，组织实施徐兰客运专线徐州段、现代化大型煤炭枢纽港、湖西航道等新建项目，积极抓好大郭庄机场搬迁、城市轨道交通等前期推进项目，拉开特大型区域性中心城市的发展框架。

　　（二）加快新城区建设。完善新城区管理运行机制和国资公司经营运作机制，突出抓好道路、景观绿化等基础设施建设，重点实施市中心医院新城区分院、新体育中心、中小学校、吉田商务广场等公共服务项目，继续推进安置房建设，积极吸引社会资本投入新城区商业开发。

　　（三）推进老城区改造。制定实施市区道路三年畅通计划，着力推进淮海路等20项道路贯通工程，建成珠江路快速通道，续建徐贾快速通道，推进淮海路省级示范路、中山路等重点道路综合整治，有效改善城市交通状况。实施云龙湖周边景观改造、彭祖园和九龙湖公园敞园改造等17项景观绿化工程。抓好供水安全保障、公交发展、垃圾收运体系建设等公用设施项目，新建或改造14个农贸市场，加快开明市场搬迁，建设一批便民疏导点。坚持依法拆迁、和谐拆迁、阳光拆迁，确保完成棚户区拆迁任务，力争城中村改造取得明显突破。

　　（四）抓好高铁站区建设。围绕建设集“交通枢纽、生态、商务、居住”四位一体的城市副中心，全面抓好高铁站区核心区建设，加快实施站房主体、地下空间和站前广场及相关主干道建设，推进山体整修复绿、长途汽车站、公交首末站及基本商业设施建设，满足京沪高铁通车需要；预留城市轨道交通通道，为后续整体开发打下良好基础。

　　（五）提高城市管理水平。进一步完善大城管的长效机制，充分发挥街道、社区在城市管理中的基础作用，切实提高城市管理??，加大社区公共服务和安全设施建设，健全社区管理体制，全面实施市区马路市场整治，推??治。建设数字化城管系统，完善主城区保洁、绿化管护市场化运作机制。

　　（六）促进房地产市场健康发展。全面贯彻落实国家和省关于房地产业发展的各项政策，合理确定房地产用地规模和供应节奏，优化区域供应结构，完善土地招拍挂和商品房预售等制度，适当增加中低价位、中小套型普通商品住房和公共租赁房用地供应，增加普通商品住房有效供给。加强商品房价格监管，研究制定房价控制措施，推行新建商品房价格备案制度，严肃查处圈地不建、捂盘惜售、哄抬房价等违法违规行为,努力遏制房价过快上涨势头。加大保障性住房建设力度，建设和购买定销安置房276万平方米，续建经济适用房及廉租房28万平方米，解决好低收入家庭的住房困难。

　　（七）推动淮海经济区核心区一体化发展。加快修编东陇海线产业带徐州区域规划，继续推动淮海经济区发展规划编制工作，着力构建大城市对应连云港大港口的发展体系。重点加强与徐州接壤的7个地市的交流合作，在交通、产业、市场、旅游等十大领域，共同探索一体化发展途径，力争淮海经济区核心区建设取得实质性进展。

　　五、全面开创和谐社会建设新格局

　　围绕建设“和谐徐州”，用更大的力度保障和改善民生，加大各项社会事业投入，努力让发展成果更多地惠及广大群众。

　　（一）全面落实为民兴办的实事项目。按照更加注重改善民生、贴近百姓的原则，坚持公共财政支出向低收入群体和弱势群体倾斜，重点实施住房保障、社会保障、民生关爱、新农村建设、蓝天碧水、生态景观、城区畅通、市场繁荣、社会事业、平安放心等10大类实事项目。我们将进一步细化落实责任、全力以赴推进，确保圆满完成各项工作任务。

　　（二）大力推动就业创业。抓好新一轮就业再就业政策的落实，积极为企业降压减负，帮助企业稳定就业岗位，推进“充分就业社区”、“充分转移村镇”创建活动，开发更多的公益性就业岗位，确保城镇“零就业家庭”、农村“零转移农户”持续“动态清零”，年内力争城镇新增就业7.5万人、失业人员再就业3.5万人。着力优化创业环境，发放创业担保贷款2亿元，扶持创业项目1000个，制定实施鼓励大学生创业的政策措施，鼓励科研人员、返乡农民工、复转军人、机关干部及退休人员创业，在全市兴起全民创业的热潮。

　　（三）不断强化社会保障。进一步加大财政投入力度，扩大社会保险覆盖面，重点促进农民工、灵活就业人员、城镇居民等群体参加城镇职工或居民社会保险。各级财政对城镇居民医疗保险和新型农村合作医疗保险的补助标准提高到每人每年120元。进一步提高社会养老保障水平，加快推进老年服务设施建设，市区9.8万名70岁以上的老年人免费乘坐公交车，新型农村社会养老保险年内基本实现全覆盖。健全社会救助机制，逐步解决59.3万残疾人生活和康复问题，继续做好17万户低保家庭及弱势群体的救助工作，努力做到应保尽保、有难必助。

　　（四）全力保障社会和谐稳定。整顿和规范市场秩序，扎实开展食品药品专项治理，抓好放心肉、放心菜等安全监管和服务体系建设，创建100家放心消费示范街道（社区）和乡镇。贯彻安全生产“一岗双责”制度，坚决杜绝重特大事故发生。完善社会突发事件应急处置体系，做好灾害救助和防灾减灾工作。健全领导干部接访下访制度，推进“阳光信访”工程建设。深入推进社会矛盾化解、社会管理创新和公正廉洁执法三项重点工作，更加重视基层基础建设，在全市建成500个规范化中心警务室；加强社会治安综合治理，健全大调解机制，构建更加严密的防控体系，严厉打击各类犯罪活动，高水平建设“平安徐州”、“法治徐州”。

　　（五）统筹发展各项社会事业。坚持教育优先发展，加快区域教育现代化进程，力争主城区、铜山县、沛县达到省教育现代化县(市、区)标准；进一步改善190万在校师生的教学和生活条件，扎实推进教师绩效工资实施工作，继续实施“中小学校舍安全”等重点工程，完成生物工程高等职业学校一期扩建、工程学院新校区二期工程建设；支持中国矿大等驻徐高校加快发展，提高科研创新和服务地方发展的能力。大力发展文化产业，培育特色文化品牌。加快市区公立医院布局调整和基本建设，以县为单位全面达到农民健康工程先进县标准。积极推进人口和计划生育综合改革，继续稳定低生育水平，促进出生人口性别比趋于平衡。认真贯彻全民健身条例，加强公共体育设施建设，精心做好省第17届运动会参赛和第18届运动会筹备工作。不断提升双拥工作水平，确保实现“全国双拥模范城”七连冠目标。全面完成对口支援四川地震灾区恢复重建任务。扎实推进精神文明建设，深入开展文明城市、社区、村镇、行业等创建活动，积极推进未成年人综合实践基地建设，大力净化社会文化环境。统筹抓好人防、民兵、民族宗教、档案、外事、侨务、台湾事务、统计、审计、广播电视、新闻出版、住房公积金管理、无线电管理、淮塔管理、气象、妇女儿童、青少年、关心下一代、老龄等方面的工作，促进各项社会事业协调发展。

　　六、进一步加强政府自身建设

　　履行好政府职责，肩负起人民重托，推动我市经济社会又好又快发展，必须按照科学发展观的要求，不断加强政府自身建设，努力提高政府及各部门的执行力。

　　（一）提高依法行政的能力。强化领导干部的宪法意识，加强行政执法人员培训，不断提高依法行政的自觉性。建立完善重大行政决策听证、合法性审查、集体决定、实施情况后评价等制度，规范行政自由裁量权行使，强化行政执法责任追究。巩固行政审批制度改革成果，深化政务公开，完善行政权力网上公开透明运行机制。坚持向市人大及其常委会报告工作，向市政协通报情况，认真办理人大代表和政协委员提出的议案、建议、提案，广泛听取各民主党派、无党派人士、工商联和人民团体的意见，积极加强对口联系，主动接受各方面的监督。

　　（二）提高为民服务的能力。始终把人民满意作为检验政府工作成效的根本标准，进一步转变机关作风，牢固树立亲民为民的鲜明工作导向。广泛开展调查研究，虚心问政于民、问需于民、问计于民，努力使工作思路、政策措施更加符合实际、符合人民群众的愿望。注重普惠民生的制度性安排，不断强化公共服务职能，建立完善各级便民服务中心，加快社区民生综合服务中心建设，尽最大努力为人民群众办好事、办实事。

　　（三）提高开拓创新的能力。以开展振兴徐州老工业基地创新奖评选活动为契机，加快培育一支“善操作、会落实、能创新”的干部队伍，努力使创新成为广大干部的基本思维方式和工作习惯。把“三重一大”作为创新的主战场、把体制机制创新作为主攻方向、把解决问题作为衡量创新成效的重要标准，大力营造崇尚创新、尊重创新、保护创新的良好环境，充分调动全市上下创新发展的积极性。以创新的精神编制“十二五”发展规划，使其成为推动我市又好又快发展的宏伟蓝图。

　　（四）提高拒腐防变的能力。始终按照“两个务必”的要求，贯彻落实中央《建立健全惩治和预防腐败体系2008-2012年工作规划》，大力加强反腐倡廉建设，深入开展反腐败斗争，不断深化“勤廉徐州”创建活动。进一步健全廉政建设制度，真正用制度管权、按制度办事、靠制度管人，从源头上预防和治理腐败；进一步增强各级干部的廉洁从政意识，严格执行厉行节约的各项规定，有效压缩行政运行成本。全面开展问责问效问廉、治散治庸治腐工作，认真做好纠风和专项治理，牢固树立为民、务实、清廉的政府形象。

　　各位代表，当前我市正处于发展的关键时期，面临的任务光荣而艰巨。让我们在党的十七大和十七届三中、四中全会精神指引下，在省委、省政府和市委的正确领导下，紧密团结和依靠全市人民，坚定信心，振奋精神，埋头苦干，开拓创新，为圆满完成“十一五”规划、加快实现全面建设小康社会目标而努力奋斗！
